# Preoperative colonoscopy in ovarian cancer: impact on surgical planning and outcomes: results from a retrospective, single-center study

**DOI:** 10.1007/s00404-025-08086-4

**Published:** 2025-06-14

**Authors:** H. Endres, D. Daneehl, S. Fichtner-Feigl, S. Huwer, D. Jakob, L. Jung, M. Klar, H. Neeff, G. Seifert, L. Yagcioglu, I. Juhasz-Boess, F.-A. Taran

**Affiliations:** 1 Department of Obstetrics and Gynecology, Faculty of Medicine, Medical Center–University of Freiburg, University of Freiburg, Freiburg, Germany; 2 Department of Women’s Health, University Hospital Tübingen, Tübingen, Germany; 3 Department of General and Visceral Surgery, Faculty of Medicine, Medical Center – University of Freiburg, Freiburg, Germany; 4 Department of Obstetrics and Gynecology, Faculty of Medicine, University Hospital Cologne, University of Cologne, Cologne, Germany

**Keywords:** Ovarian cancer, Preoperative colonoscopy, Surgical outcomes, Colorectal involvement, Interdisciplinary coordination

## Abstract

**Background:**

Diagnosis and management of ovarian cancer remain complex due to the overlap of symptoms with other malignancies and the variability in preoperative diagnostic approaches. While histological confirmation is crucial, the role of preoperative colonoscopy in improving surgical planning and patient outcomes remains unclear.

**Objective:**

This study aims to evaluate the impact of preoperative colonoscopy on surgical outcomes, peri-operative complications and interdisciplinary coordination in ovarian cancer patients.

**Methods:**

A retrospective, single-center study was conducted at the University Medical Center Freiburg, including 306 patients diagnosed with malignant ovarian tumors between 2016 and 2023. Patients were stratified into two groups: those who underwent preoperative colonoscopy (*n*=104) and those who did not (*n*=202). Tumor characteristics, diagnostic findings, and surgical outcomes were compared. Primary endpoints included the detection of abnormal colonoscopic findings and their correlation with intraoperative interventions. Secondary endpoints assessed the impact of colonoscopy on macroscopic complete resection rates and peri-operative complications.

**Results:**

Patients undergoing preoperative colonoscopy exhibited higher rates of advanced tumor stages (FIGO III/IV: 84.5% vs. 47.5%). Abnormal colonoscopic findings were observed in 38.8% of cases, yet colorectal resections were performed in only 53% of these patients. Despite a higher frequency of neoadjuvant chemotherapy in the colonoscopy group (57.3 vs. 33.7%), macroscopic complete resection rates were lower (67.0 vs. 79.2%). Sensitivity and specificity analyses indicated moderate predictive accuracy of colonoscopy for colorectal involvement (67 and 74%, respectively). In advanced ovarian cancer, preoperative colonoscopy influenced colorectal surgery decisions, with higher resection rates but minimal impact on neoadjuvant chemotherapy rates, despite moderate sensitivity and specificity.

**Conclusion:**

While preoperative colonoscopy identified colorectal involvement in a subset of ovarian cancer patients, particularly in advanced tumor stages, its impact on surgical decision-making, oncological outcomes, and physicians' choice for neoadjuvant chemotherapy was limited. The findings suggest that intraoperative assessments remain the primary determinant for colorectal interventions. Future prospective studies are warranted to clarify the clinical utility of colonoscopy in preoperative evaluation and its potential influence on interdisciplinary surgical strategies.

**Retrospectively registered study:**

24-1364-S1-retro

## Take-home message

**Table:** 

This study highlights the limited clinical value of routine properative colonoscopy in ovarian cancer patient, showing moderate diagnostic accuracy and minimal impact on surgical outcomes or treatment planning. Intraoperative assesment remains the key determinant for colorectal interventions, suggesting colonoscopy should be reserved for select cases with clear indications.	

## Introduction

Ovarian cancer remains a challenging disease to diagnose and treat due to variations and inconsistencies in diagnostic practices. The primary goal in suspected cases is to determine tumor type and assess operability. While advancements in preoperative diagnostic tools have improved initial assessments, histological confirmation remains essential as conditions such as colorectal carcinoma can mimic both symptoms and appearance of ovarian cancer [1]. In advanced ovarian cancer, achieving macroscopic complete resection is a key factor in improving overall survival [1–3]. However, the impact of preoperative diagnostic tools on survival outcomes remains limited, often providing no significant advantage over intraoperative assessments [4, 5]. Preoperative evaluations vary in their invasiveness, duration, and associated morbidity. Common approaches include radiological imaging, diagnostic laparoscopy, and colonoscopy, with diagnostic laparoscopy shown to enhance the likelihood of successful macroscopic resection [6]. In cases where complete resection is unlikely, neoadjuvant chemotherapy followed by interval debulking is preferred as it reduces peri-operative complications, increases the chances of complete resection, and does not affect long-term survival [2, 3, 7, 8].

Although invasive, preoperative colonoscopy and gastroscopy allow for the assessment of the stomach, duodenum, rectum, sigmoid, and colon, aiding in the exclusion of colorectal or gastric carcinomas [9–11]. Studies estimate that 4–6% of ovarian cancer patients have concomitant colorectal or gastric metastases detectable through these procedures [12, 13]. However, routine colonoscopy screening presents challenges, including preparation time that may delay surgery, increased risks in older or comorbid patients, and inability to evaluate the small intestine despite its frequent involvement in advanced ovarian cancer [11, 14–19]. Furthermore, preoperative colonoscopy is often performed to facilitate coordination between gynecological and visceral surgeons for complex cytoreductive procedures [17, 20]. Evidence suggests that gynecological oncologists alone may achieve lower stoma placement and mortality rates [21], and the role of colonoscopy in improving interdisciplinary coordination and oncological outcomes remains uncertain.

Given the lack of European data and standardized guidelines, this retrospective single-center study aims to evaluate the impact of preoperative colonoscopy on surgical outcomes, peri-operative complications, and interdisciplinary coordination in ovarian cancer patients. Our study focuses on assessing whether colonoscopy influences colorectal resection rates, neoadjuvant chemotherapy administration, and macroscopic complete resection, thereby clarifying its clinical utility in preoperative planning.

## Methods

This single-center, retrospective study was conducted at the University Medical Center Freiburg and included patients aged 18 years or older who were diagnosed with malignant ovarian tumors between January 1, 2016, and December 31, 2023. Eligibility criteria required a histologically confirmed malignant ovarian tumor and treatment with curative surgical intent.

Data for this study were extracted from the internal hospital database following approval from the Ethics Committee of the University of Freiburg (approval number: 24-1364-S1-retro). Key variables included preoperative diagnostic procedures, tumor stage according to the Federation of Gynecology and Obstetrics (FIGO) 2008 staging system, and the type of surgical intervention, categorized as either primary cytoreductive surgery or interval debulking following neoadjuvant chemotherapy. Preoperative colonoscopy results were classified as normal or abnormal, with abnormal findings defined as endoscopic evidence of tumor-related compression or perforation involving the colon, sigmoid, or rectum.

This study adhered to the principles outlined in the Declaration of Helsinki and complied with all relevant national and international ethical guidelines. Patients were divided into two groups based on whether they had undergone preoperative colonoscopy. This study group consisted of patients who underwent the procedure, while the comparison group included those who did not. Additionally, a subgroup analysis was conducted for patients with advanced ovarian cancer, specifically those classified as FIGO stages III and IV.

The primary study endpoints were colonoscopic findings and the performance of colorectal resection or shaving during cytoreductive surgery. Secondary endpoints included the detection of secondary carcinomas during colonoscopy and the achievement of macroscopic complete resection.

To ensure sufficient statistical power, this study aimed to include approximately 100 patients who underwent diagnostic colonoscopy. Descriptive analysis was performed for both study groups, and colonoscopy results as well as intraoperative interventions were recorded as categorical variables. Sensitivity and specificity of preoperative colonoscopy in predicting the need for colorectal resections were calculated, and categorical variables were analyzed using the chi-square test. Data collection and statistical analysis were conducted using Microsoft Excel (Version 2024) [Microsoft Corporation, 2024] and IBM SPSS Statistics (Version 29.0, IBM Corp., Armonk, NY).

## Results

Our comprehensive study of 306 patients with ovarian cancer provided nuanced insights into the role of preoperative colonoscopy. The patient cohort, with a mean age of 59.5 years, underwent surgical treatment between 2016 and 2023, with 34% (104 patients) receiving preoperative colonoscopy.

Analysis of tumor characteristics and diagnostic findings revealed that patients in the colonoscopy group presented with more advanced disease stages. Among these patients, the proportion of FIGO stage III cases increased from 29.7 to 53.4%, while FIGO stage IV cases rose from 17.8 to 31.1%. Notably, abnormal colonoscopic findings were observed in 38.8% of cases, with the prevalence varying across tumor stages. Specifically, abnormal findings were detected in 18.2% of FIGO stage I patients, 33.3% of FIGO stage II patients, 50.9% of FIGO stage III patients, and 28.1% of FIGO stage IV patients (Table 1).

**Table 1:** Characteristics of the study and control group. NACT= neoadjuvant chemotherapy

	Study group (*n*=104)	Control group (*n*=202)
Age (in years)	Mean: 62.00	Mean: 58.35
Tumor stage		
FIGO I	10.7% (*n*=11 )	33.2% (*n*=67 )
FIGO II	2.9% (*n*=3 )	9.4% (*n*=19 )
FIGO III	53.4% (*n*=55 )	29.7% (n=60 )
FIGO IV	31.1% (*n*=32 )	17.8% (*n*=36 )
Unknown	1.9% (*n*=2 )	9.9% (*n*=20)
Histology		
High-grade serous ovarian carcinoma	85.7% (*n*=66 )	66.1% (*n*=72)
Mucinous ovarian carcinoma	3.9% (*n*=3 )	14.7% (*n*=16)
Endometrioid ovarian carcinoma	6.5% (*n*=5 )	12.8% (*n*=14)
Clear cell ovarian carcinoma	3.9% (*n*=3 )	6.4% (*n*=7)
NACT performed	57.3% (*n*=59 )	33.7% (*n*=68)
Colonoscopy Findings		
Abnormality	38.8% (*n*=40 )	0.0% (*n*=0 )
Colorectal resection/shaving		
Resection	32.0% (*n*=33 )	15.3% (*n*=31 )
Shaving	3.9% (*n*=4 )	1.5% (*n*=3 )
Inoperability	21.4% (*n*=22 )	14.4% (*n*=29 )
No infestation (appendectomy for muc. carcinoma if necessary)	42.7% (*n*=44 )	68.8% (*n*=139 )
Postoperative tumor residue		
Macroscopic complete resection	67.0% (*n*=69)	79.2% (*n*=160 )
No macroscopic complete resection	34.0% (*n*=35)	20.8% (*n*=42 )

Patients who underwent preoperative colonoscopy experienced distinct treatment pathways. They were more likely to receive neoadjuvant chemotherapy, with rates of 57.3% compared to 33.7% in those who did not undergo colonoscopy. Additionally, these patients had a lower likelihood of achieving macroscopic complete resection, with rates of 67.0% versus 79.2%. The frequency of colorectal resection was also notably higher in the colonoscopy group, occurring in 32.0% of cases compared to 15.3% in patients who did not undergo the procedure (Table 1).

**Fig. 1:**
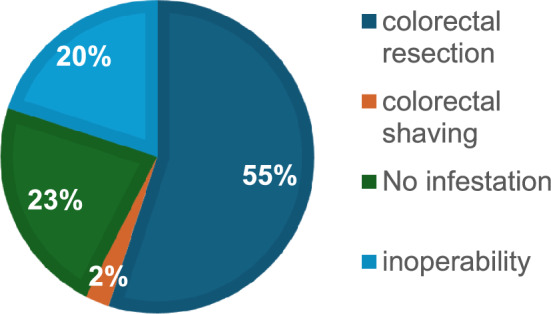
Study group analysis: Colorectal surgery in patients with abnormal colonoscopy finding

**Fig. 2:**
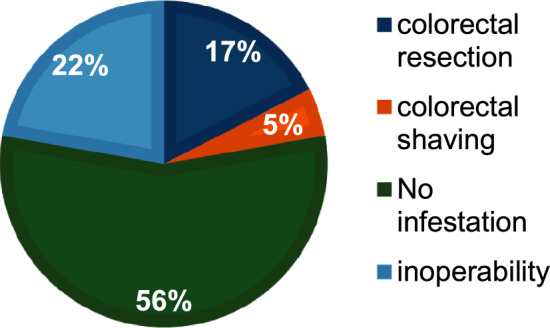
Study group analysis: Colorectal surgery in patients with normal colonoscopy finding

In evaluating the diagnostic performance of preoperative colonoscopy, sensitivity was calculated at 67%, while specificity was 74%. Colorectal resection was performed in 55% of cases with abnormal colonoscopy findings, whereas 17% of patients with unremarkable colonoscopic results still required surgical colorectal intervention (Appendix: Fig. 1 and Fig. 2). Among patients with abnormal colonoscopy findings, 55% underwent colorectal resection, 2% underwent colorectal shaving, 23% showed no evidence of colorectal infestation upon surgical exploration, and 20% were deemed inoperable (Appendix: Figure 1). In contrast, for patients with normal colonoscopy results, 17% underwent colorectal resection, 5% underwent colorectal shaving, 56% had no identifiable colorectal infestation, and 22% were classified as inoperable (Appendix: Figure 2). The chi-square test yielded a statistic of 15.42 (*p* = 0.000086), indicating a statistically significant but limited association between colonoscopy findings and the need for colorectal surgery.

Among the subgroup of 183 patients with advanced ovarian cancer, colonoscopy revealed significant diagnostic and treatment patterns. Stage distributions were comparable between groups, with abnormal findings identified in 42.5% of examinations. Neoadjuvant chemotherapy rates remained similar, at 65.5% in the colonoscopy group compared to 60.4% in the non-colonoscopy group. Macroscopic complete resection rates were also consistent between the two groups, at 63.2% and 63.5%, respectively. However, the frequency of colorectal resection was higher in patients who underwent colonoscopy, with rates of 36.8% compared to 27.1% (Table 2).

**Table 2:** Characteristics of the subgroup analysis for advanced ovarian cancer stages (FIGO III and IV) study and control group

Advanced ovarian cancer group	Study Group (*n*=87)	Control Group (*n*=96)
Age (in years)	mean: 62.83	mean: 64.17
Tumor stage		
FIGO III	63.2% (*n*=55 )	62.5% (*n*=60 )
FIGO IV	36.8% (*n*=32 )	37.5% (*n*=36 )
Histology		
High-grade serous ovarian carcinoma	93.8% (*n*=60 )	90.0% (*n*=54 )
Mucinous ovarian carcinoma	1.6% (*n*=1 )	0.0% (*n*=0 )
Endometrioid ovarian carcinoma	1.6% (*n*=1 )	3.3% (*n*=2 )
Clear cell ovarian carcinoma	3.1% (*n*=2 )	6.7% (*n*=4 )
NACT performed	65.5% (*n*=57 )	60.4% (*n*=58 )
Colonoscopy Findings		
Abnormality	42.5% (*n*=37 )	
Colorectal resection/shaving		
Resection	36.8% (*n*=32)	27.1% (*n*=26)
Shaving	4.6% (*n*=4)	2.1% (*n*=2 )
Inoperability	25.3% (*n*=22)	27.1% (*n*=26)
No infestation (appendectomy for muc. carcinoma if necessary)	33.3% (*n*=29)	42.7% (*n*=41)
Postoperative tumor residue		
Macroscopic complete resection	63.2% (*n*=55)	63.5% (*n*=61)
No macroscopic complete resection	36.8% (*n*=32)	36.5% (*n*=35)

Among the 183 patients with advanced ovarian cancer, 57.5% exhibited evidence of rectal, sigmoid, or colonic involvement, including cases where surgery was canceled due to excessive tumor burden. Colorectal interventions were more frequently performed in patients who underwent preoperative colonoscopy (Table 2). Sensitivity and specificity of colonoscopy in this subgroup were 54% and 76%, respectively.

**Fig. 3:**
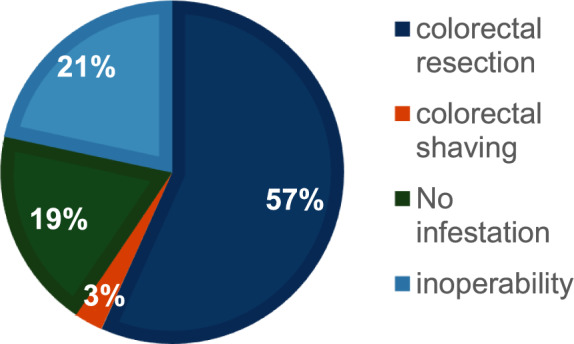
Study subgroup analysis: Colorectal surgery in patients with abnormal colonoscopy finding

**Fig. 4:**
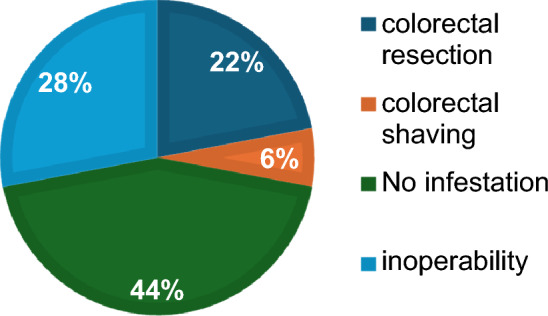
Study subgroup analysis: Colorectal surgery in patients with normal colonoscopy finding

Surgical decision-making varied according to colonoscopic findings. Among patients with abnormal colonoscopy results, 57% underwent colorectal resection, 3% underwent colorectal shaving, 19% had no evidence of colorectal infestation at surgery, and 21% were deemed inoperable (Appendix: Fig. 3). In the group with normal colonoscopy findings, 22% underwent colorectal resection, 6% underwent shaving, 44% showed no intraoperative evidence of colorectal involvement, and 28% were considered inoperable (Appendix: Fig. 4). The chi-square test yielded a statistic of 6.72 (*p* = 0.00965), indicating a statistically significant but limited association between colonoscopy findings and the need for colorectal surgery.

## Discussion

Our findings align with existing literature regarding the high prevalence of advanced ovarian cancer at primary diagnosis [22]. Colonoscopy was performed in 34% of patients with ovarian tumors, with increased utilization in those with advanced disease stages. Younger patients with advanced disease underwent colonoscopy more frequently, potentially indicating a selection bias favoring optimal tumor resection in this subgroup. To our knowledge, no objective criteria were applied to determine the necessity of preoperative colonoscopies. The study group exhibited a higher proportion of advanced tumor stages, suggesting that colonoscopies were primarily performed to assess tumor resectability rather than to detect secondary colorectal malignancies. If colonoscopy were routinely used for carcinoma detection, its distribution across all tumor stages would likely be more balanced [22, 23]. Given this trend, clinicians in the study group anticipated a lower likelihood of achieving macroscopic complete resection and were more likely to initiate neoadjuvant chemotherapy [24]. This perception was likely influenced by the higher proportion of advanced tumor stages in this cohort.

Contrary to previous studies, no secondary colorectal carcinomas were detected or documented in our cohort [11, 12]. This discrepancy may be attributed to our data collection methodology, which was based on coded primary tumor diagnoses. As a result, patients ultimately diagnosed with colorectal cancer rather than ovarian cancer may have been excluded from our dataset. In the subgroup of patients with advanced ovarian cancer, neoadjuvant chemotherapy rates were comparable between the study and the comparison groups. Colonoscopy abnormalities were identified in 42.5% of cases but did not significantly influence the decision to administer neoadjuvant chemotherapy. Previous studies have reported abnormal colonoscopy findings in 12% to 56.9% of ovarian cancer patients [11, 12]. During surgery, a higher rate of colorectal interventions was observed in patients who underwent preoperative colonoscopy, which aligns with the increased prevalence of advanced disease in this group [22, 23]. However, in the subgroup of patients with advanced ovarian cancer, a higher rate of colorectal resections was observed in those who had undergone preoperative colonoscopy, a finding that cannot be fully explained by differences in disease severity alone. Despite the higher rate of neoadjuvant chemotherapy in the study group, colorectal resection rates remained elevated, possibly due to increased awareness of surgical complexity and improved preoperative preparation. While previous studies have suggested lower colorectal resection rates following neoadjuvant chemotherapy [25], the performance of colonoscopy and subsequent abnormal findings may contribute to higher rates of visceral surgical involvement in cytoreductive procedures, leading to increased rates of stoma creation and colorectal resections [21].

Importantly, colorectal resection was performed in only 53% of cases with abnormal colonoscopy findings, while 18% of patients with inconspicuous findings still required colorectal surgery. This indicates that intraoperative decisions regarding colorectal interventions are not solely guided by preoperative colonoscopic findings. Notably, macroscopic complete resection rates remained consistent across different diagnostic approaches, suggesting that preoperative colonoscopy did not significantly impact fundamental surgical outcomes. The extent to which preoperative preparation based on colonoscopy findings influences resection rates remains unclear.

A key strength of this study is the large sample size and the inclusion of a comprehensive subgroup analysis for advanced tumor stages. Furthermore, this study contributes European data to a previously underexplored clinical question. Limitations include its retrospective nature, lack of standardized criteria for recommending colonoscopy, and potential selection bias. The single-center design may also limit generalizability.

These findings underscore the limited predictive value of colonoscopy for surgical planning and highlight the central role of intraoperative assessment. The routine use of preoperative colonoscopy should be critically evaluated, particularly in the absence of standardized criteria for its application. While potentially reducing the need for on-call visceral surgeons during cytoreductive surgery is promising for optimizing healthcare resource utilization, its clinical benefits must be carefully weighed. Future research can enhance ovarian cancer management by refining the role of preoperative colonoscopy. Providing feedback to gastroenterologists may improve diagnostic accuracy, while prospective studies on its impact on surgical outcomes, overall survival, and secondary carcinoma detection could offer valuable insights.

## Conclusion

This study demonstrates that preoperative colonoscopy in ovarian cancer patients was more frequently performed in cases with advanced disease and associated with higher rates of colorectal interventions. However, colonoscopic findings did not reliably predict the need for colorectal surgery, and no improvement in macroscopic complete resection rates was observed. These findings underscore the limited role of colonoscopy in preoperative decision-making, with intraoperative assessment remaining the key determinant for surgical planning. Routine colonoscopy should be reconsidered unless clear, standardized indications are established.

## Data Availability

No datasets were generated or analysed during the current study.

## Colorectal surgery in patients with abnormal colonoscopy findings

See Fig. 1

## Colorectal surgery in patients with normal colonoscopy findings

See Fig. 2

## Advanced ovarian cancer: colorectal surgery in patients with abnormal colonoscopy findings

See Fig. 3

## Advanced ovarian cancer: colorectal surgery in patients with normal colonoscopy findings

See Fig. 4
